# Excess mortality associated with the COVID-19 pandemic among Californians 18–65 years of age, by occupational sector and occupation: March through November 2020

**DOI:** 10.1371/journal.pone.0252454

**Published:** 2021-06-04

**Authors:** Yea-Hung Chen, Maria Glymour, Alicia Riley, John Balmes, Kate Duchowny, Robert Harrison, Ellicott Matthay, Kirsten Bibbins-Domingo

**Affiliations:** 1 Institute for Global Health Sciences, University of California, San Francisco, California, United States of America; 2 Department of Epidemiology and Biostatistics, University of California, San Francisco, California, United States of America; 3 Department of Medicine, University of California, San Francisco, California, United States of America; Sciensano, BELGIUM

## Abstract

**Background:**

Though SARS-CoV-2 outbreaks have been documented in occupational settings and in-person essential work has been suspected as a risk factor for COVID-19, occupational differences in excess mortality have, to date, not been examined. Such information could point to opportunities for intervention, such as vaccine prioritization or regulations to enforce safer work environments.

**Methods and findings:**

Using autoregressive integrated moving average models and California Department of Public Health data representing 356,188 decedents 18–65 years of age who died between January 1, 2016 and November 30, 2020, we estimated pandemic-related excess mortality by occupational sector and occupation, with additional stratification of the sector analysis by race/ethnicity. During these first 9 months of the COVID-19 pandemic, working-age adults experienced 11,628 more deaths than expected, corresponding to 22% relative excess and 46 excess deaths per 100,000 living individuals. Sectors with the highest relative and per-capita excess mortality were food/agriculture (39% relative excess; 75 excess deaths per 100,000), transportation/logistics (31%; 91 per 100,000), manufacturing (24%; 61 per 100,000), and facilities (23%; 83 per 100,000). Across racial and ethnic groups, Latino working-age Californians experienced the highest relative excess mortality (37%) with the highest excess mortality among Latino workers in food and agriculture (59%; 97 per 100,000). Black working-age Californians had the highest per-capita excess mortality (110 per 100,000), with relative excess mortality highest among transportation/logistics workers (36%). Asian working-age Californians had lower excess mortality overall, but notable relative excess mortality among health/emergency workers (37%), while White Californians had high per-capita excess deaths among facilities workers (70 per 100,000).

**Conclusions:**

Certain occupational sectors are associated with high excess mortality during the pandemic, particularly among racial and ethnic groups also disproportionately affected by COVID-19. In-person essential work is a likely venue of transmission of coronavirus infection and must be addressed through vaccination and strict enforcement of health orders in workplace settings.

## Introduction

More deaths are occurring during the COVID-19 pandemic than predicted by historical trends [1–4]. In California, which recorded more than 1.2 million COVID-19 cases and 19,000 deaths through November 2020, per-capita excess mortality is relatively high among Blacks, Latinos, and individuals with low educational attainment [4]. Workplace settings have been postulated as a risk factor for COVID-19 and may be related to these documented disparities, but whether excess mortality varies across occupation has not been fully examined. We are particularly interested in so-called essential workers, since such individuals have been categorically exempted from shelter-in-place orders and often cannot work from home [4–6]. Such information could point to opportunities for intervention among occupational groups facing heightened transmission risks, such as workplace modifications and prioritization of vaccine distribution. Using autoregressive integrated moving average models to forecast deaths from March through November 2020, we compare excess deaths among California residents 18–65 years of age across occupational sectors and occupations, with additional stratification of the sector analysis by race/ethnicity. To explore differences across time, we additionally estimated excess mortality for March–May, June–August, and September–November. Accounting for lags from policy to infection to death these roughly correspond, respectively, to a period of sheltering in place, a period of business reopenings, and a period of business closures. Proportional increases in mortality compared to expected may conceal differential impacts on groups that were at elevated mortality risk prior to the pandemic; we therefore describe both relative (percent) excess in mortality and absolute (per capita) excess deaths within occupational and racial/ethnic subgroups.

## Methods

We obtained data from the California Department of Public Health on all deaths occurring on or after January 1, 2016. Our study was specifically approved by the State of California Committee for the Protection of Human Subjects. In total, we analyzed data representing 356,188 decedents 18–65 years of age.

To focus on individuals whose deaths were most plausibly linked to work, and because the US retirement age for full benefits under Social Security is 66, we restricted our analysis to decedents 18–65 years of age. Death certificates include an open text field for “Decedent’s usual occupation,” described as “type of work done during most of working life.” Retirement is not separately recorded. We processed the occupation information listed on the death certificates using the National Institute for Occupational Safety and Health’s Industry & Occupation Computerized Coding System (NIOCCS), a machine-learning system checked for consistency and accuracy that converts free-text occupational data to 2010 US Census codes. A team of 3 researchers manually categorized the resulting 529 unique codes into occupational sectors, using the 13 sectors identified by California officials as comprising the state’s essential workforce [7], plus an additional category for retail workers. Under the state’s designations, not all occupations within a sector are necessarily essential. For example, the state’s definition of the essential workforce within manufacturing lists 5 criteria. We attempted to adhere to the criteria as much as possible, but in many cases the information available in the data were not sufficiently detailed to permit this. Thus, the categorizations are, in practice, primarily based on the sectors identified by the state, rather than the specific criteria for each sector. Further information on this matter is provided in the Discussion. To ease presentation, we combined or eliminated some sectors, placing the defense, communications/IT, and financial sectors in the not-essential category (under the logic that it was particularly difficult to ascertain which workers in these sectors fully met the state’s definitions for essential work) and placing chemical, energy, and water sectors in the facilities category. This resulted in the following 9 groups: facilities, food/agriculture, government/community, health/emergency, manufacturing, retail, transportation/logistics, not essential, and unemployed/missing. Known homemakers, retirees, and students were classified as unemployed/missing. We defined 4 racial/ethnic groups: Asian, Black, Latino, and White, with the definition of Latino overwriting any racial designation in the death records. Our analyses involving race/ethnicity excluded 13,805 decedents who were multiracial or not otherwise identified as one of the 4 racial/ethnic groups defined above. Because of the existence of the unknown/missing category for sector, no decedent was excluded in sector-only analyses due to missingness of sector.

The time period of interest is March 1, 2020 through November 30, 2020. In some time-stratified analysis, we compared the months of March–May, June–August, and September–November. Accounting for lags from policy to infection to death, the cutoffs roughly correspond to major policy decisions. Specifically: the state issued a shelter-in-place order on March 19, announced reopenings of certain businesses on May 7, and announced closure of certain businesses (including bars and indoor restaurants) on July 13.

We conducted time-series analysis for each occupational sector, with additional stratification by race/ethnicity. For each group of interest (for example, each occupational sector of interest), we repeated the following procedure. We aggregated the data to months or weeks, using the weekly analysis for visualizations and the monthly analysis to derive summary measures. Following our previous work [4], we fit dynamic harmonic regression models with autoregressive integrated moving average (ARIMA) errors for the number of monthly/weekly all-cause deaths, using deaths occurring among the group between January 1, 2016 and March 1, 2020. For each iteration, we used a model-fitting procedure described by Hyndman and Khandakar [8]. Using the final model, we forecast the number of deaths for each unit of time, along with corresponding 95% prediction intervals (PI). To obtain the total number of excess deaths for the entire time window, we subtracted the total number of expected (forecast) deaths from the total number of observed deaths. We obtained a 95% PI for the total by simulating the model 10,000 times, selecting the 97.5% and 2.5% quantiles, and subtracting the total number of observed deaths.

In addition to the estimated number of excess deaths, we calculated and report per-capita excess deaths: the observed number of deaths minus the expected number of deaths, divided by the population size. We obtained population sizes from the 2019 American Community Survey. We also calculated and report the observed number of deaths divided by the expected number of deaths. These ratios represent relative excess mortality. For example, a ratio of 1.5 would indicate that there were 50% more deaths observed during the pandemic than we would have expected had the pandemic not occurred. For both measures, the comparison is between the pandemic and non-occurrence of the pandemic. The reference group is non-occurrence of the pandemic.

We also estimated excess mortality for all 529 unique occupation codes (see above). For this analysis, we projected 2020 deaths by taking the arithmetic mean of 2018 and 2019 deaths. We use two years pre-pandemic data in this analysis to minimize influence from earlier years (and because this analysis does not include modeling of time).

We conducted all analyses in R, version 4.04.

## Results

We estimate that from March 2020 through November 2020, there were 11,628 (95% PI: 10,779–12,468) excess deaths among Californians 18–65 years of age (Table 1), corresponding to relative excess of 22% (95% PI: 20–24%) and 46 (95% PI: 43–49) excess deaths per 100,000 living individuals. Half of the excess deaths are attributable to officially recorded COVID-19 deaths. Relatively large numbers of excess deaths were recorded among workers in the facilities (2,119; 95% PI: 1,711–2,518) and transportation/logistics sectors (1,649; 1,453–1,842).

**Table 1:** Excess mortality among Californians 18–65 years of age, by occupational sector, March through November 2020.

	Excess deaths	COVID-19 deaths	Per-capita excess^a^	Relative excess^b^
Entire state	11,628 (10,779–12,468)	5,813	46 (43–49)	1.22 (1.20–1.24)
Facilities	2,119 (1,711–2,518)	1,093	83 (67–98)	1.23 (1.18–1.29)
Food or agriculture	1,424 (1,248–1,596)	691	75 (66–85)	1.39 (1.32–1.45)
Government or community	567 (459–673)	328	24 (20–29)	1.17 (1.13–1.20)
Health or emergency	611 (541–680)	395	30 (27–34)	1.17 (1.15–1.19)
Manufacturing	700 (662–738)	539	61 (57–64)	1.24 (1.23–1.26)
Retail	601 (521–678)	263	38 (33–43)	1.21 (1.18–1.24)
Transportation or logistics	1,649 (1,453–1,842)	772	91 (81–102)	1.31 (1.26–1.36)
Not essential	1,335 (1,077–1,590)	744	17 (14–20)	1.12 (1.09–1.14)
Unemployed or missing	2,397 (2,139–2,653)	988	59 (52–65)	1.25 (1.22–1.28)

^a^ Defined as the observed number of deaths minus the expected number of deaths, divided by the population size, and multiplied by 100,000. The measure compares the pandemic to the counterfactual non-occurrence of the pandemic, as modeled using pre-pandemic data. The reference groups are the same groups of interest, under the counterfactual exposure.

^b^ Defined as the observed number of deaths divided by the expected number of deaths. The measure compares the pandemic to the counterfactual non-occurrence of the pandemic, as modeled using pre-pandemic data. The reference groups are the same groups of interest, under the counterfactual exposure.

Per-capita excess mortality was highest among transportation/logistics workers (91 per 100,000; 95% PI: 81–102), facilities workers (83; 95% PI: 67–98), food/agriculture workers (75; 95% PI: 66–85), and manufacturing workers (61; 95% PI: 57–64). Similarly, relative excess was highest among food/agriculture workers (39%; 95% PI: 32–45%) and transportation/logistics workers (31%; 95% PI: 26%–36%). Excess mortality among Californians in non-essential sectors was lower in both relative (12%; 95% PI 9–14) and per-capita measures (17 excess deaths per 100,000; 95% PI 14–20).

**Fig 1:**
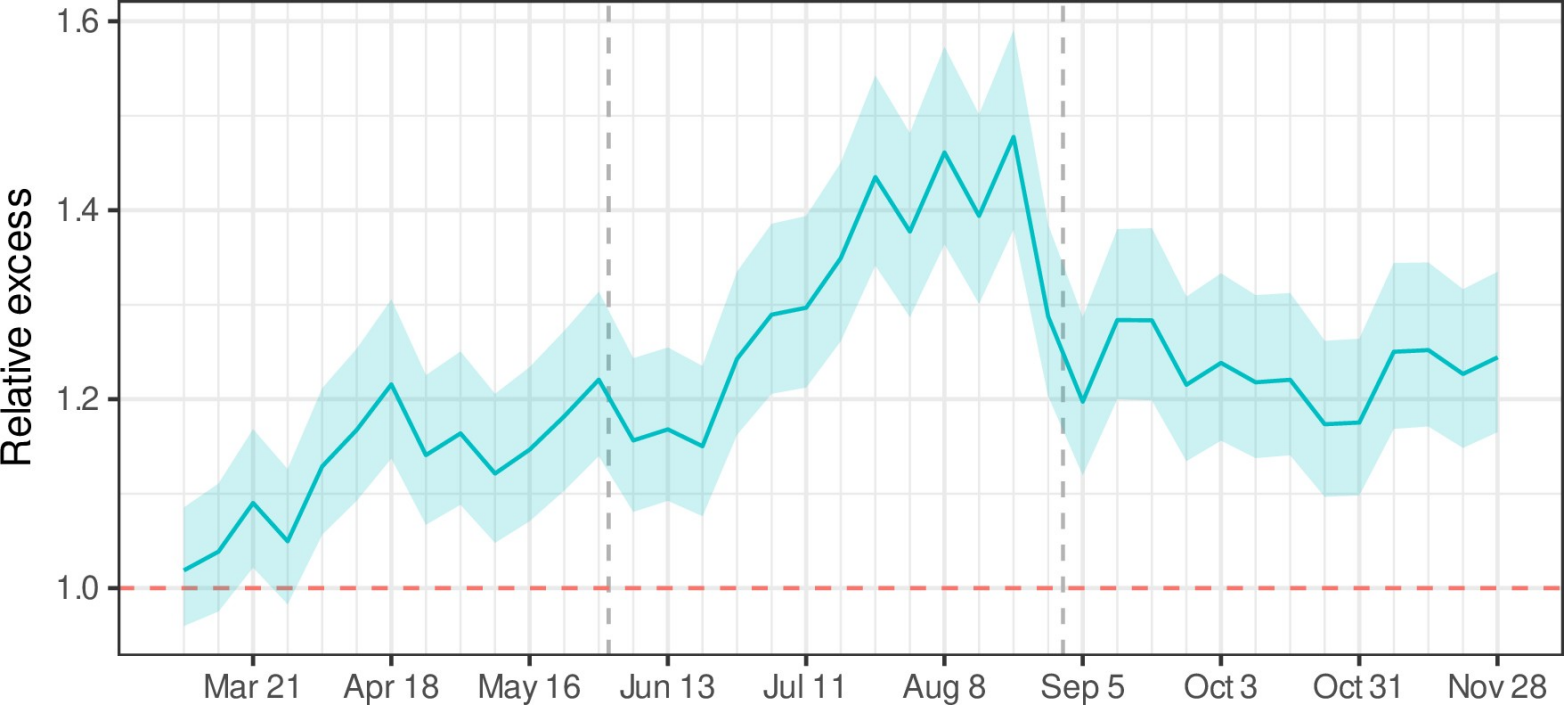
Relative excess mortality among Californians 18–65 years of age, March through November 2020. The dashed vertical lines separate the first 3 quarters of the first year of the COVID-19 pandemic. The quarters roughly correspond to major policy decisions, after accounting for lags from policy decisions to infection to death. The first quarter corresponds to a period of sheltering in place, the second quarter corresponds to a period of reopening, and the third quarter corresponds to a period of business closures.

**Fig 2:**
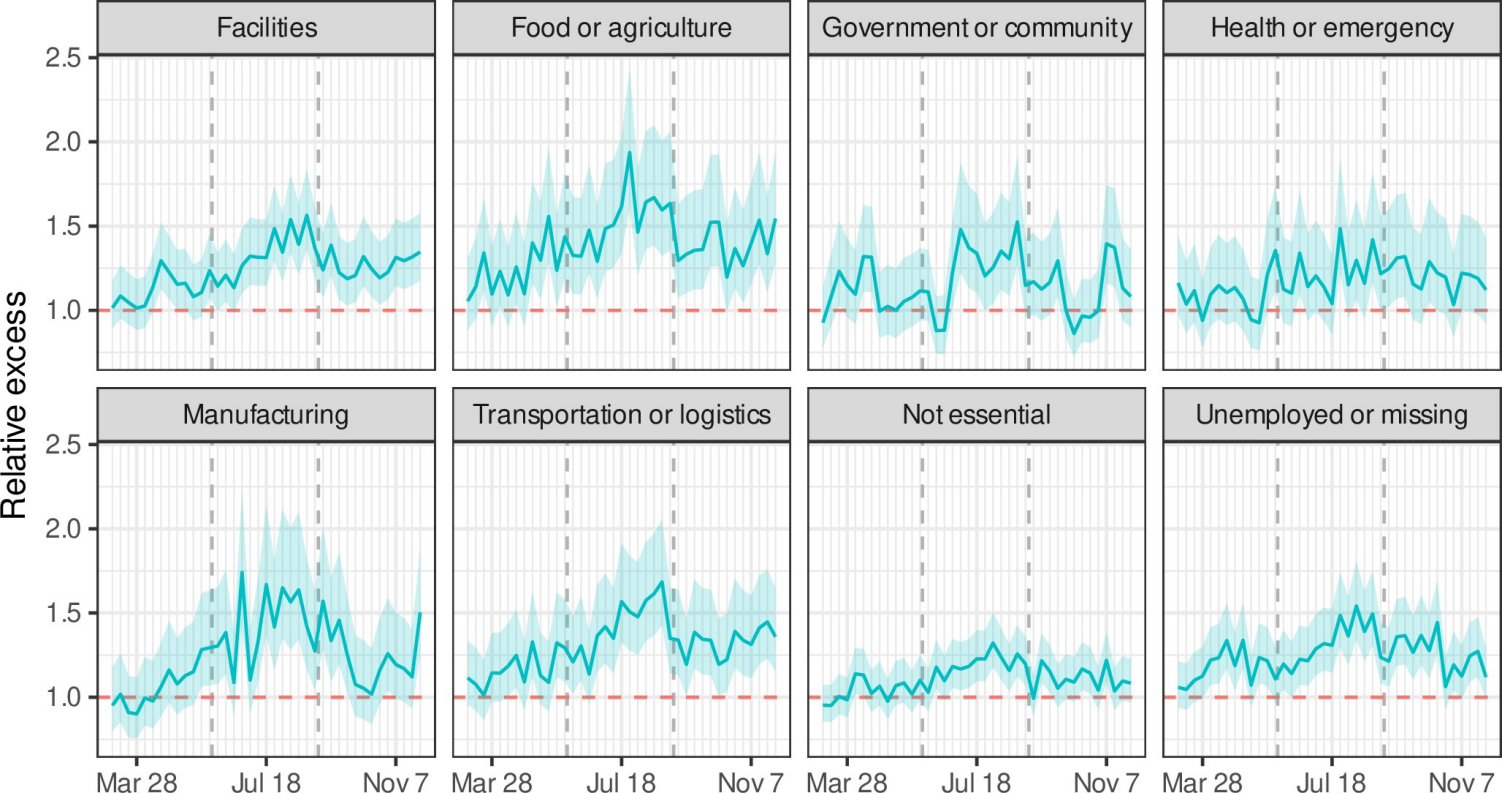
Relative excess mortality among Californians 18–65 years of age, by occupational sector, March through November 2020. The dashed vertical lines separate the first 3 quarters of the first year of the COVID-19 pandemic. The quarters roughly correspond to major policy decisions, after accounting for lags from policy decisions to infection to death. The first quarter corresponds to a period of sheltering in place, the second quarter corresponds to a period of reopening, and the third quarter corresponds to a period of business closures.

Excess mortality varied over time (Fig 1), with relative excess of 13% between March and May (95% PI: 10–16%), 32% between June and August (95% PI: 28–35%), and 23% between September and November (20–27%). These trends varied by occupational sector (Fig 2), with particularly high June–August relative excess among food/agriculture (52%; 95% PI: 43–63%), manufacturing (44%; 95% PI: 38–51%), and transportation/logistics (43%; 95% PI: 35–53%) workers.

Excess mortality also varied by race/ethnicity (Tables 2 and 3). Latino Californians had the highest relative excess mortality as well as high per-capita excess (37%, 95% PI: 30–45%; 61 excess deaths per capita 95% PI 51–70), with particularly high per-capita excess among food/agriculture workers (97; 95% PI: 65–101), transportation/logistics workers (97; 95% PI: 80–114), and manufacturing workers (96; 95% PI: 86–105). Per-capita excess mortality was highest among Black Californians at 110 excess deaths per capita (95% PI: 93–126), with relative excess of 28% (95% PI: 22–33%). Black Californians had high relative and per-capita excess among transportation/logistics workers (36%; 95% PI: 27–45%; 188 excess deaths per capita 95% PI 153–222) and high per-capita excess among facilities workers (252; 95% PI: 188–315). Asian Californians had lower relative and per-capita excess mortality but high relative excess among health/emergency workers (37%; 95% PI: 31–43). White Californians had notably high per-capita excess among facilities workers (70; 95% PI: 35–103).

**Table 2:** Relative excess mortality among California residents 18–65 years of age, by occupational sector and race/ethnicity, March through November 2020.

	All races	Asian	Black	Latino	White
All sectors	1.22 (1.20–1.24)	1.11 (1.03–1.20)	1.28 (1.22–1.33)	1.37 (1.30–1.45)	1.08 (1.03–1.13)
Facilities	1.23 (1.18–1.29)	1.21 (1.08–1.39)	1.29 (1.20–1.38)	1.35 (1.26–1.46)	1.12 (1.06–1.19)
Food or agriculture	1.39 (1.32–1.45)	1.24 (1.13–1.38)	1.32 (1.15–1.55)	1.59 (1.48–1.72)	1.13 (1.06–1.21)
Government or community	1.17 (1.13–1.20)	1.19 (1.05–1.36)	1.19 (1.09–1.32)	1.45 (1.36–1.56)	1.02 (0.94–1.10)
Health or emergency	1.17 (1.15–1.19)	1.37 (1.31–1.43)	1.23 (1.14–1.34)	1.33 (1.20–1.47)	1.01 (0.95–1.08)
Manufacturing	1.24 (1.23–1.26)	1.21 (1.10–1.35)	1.17 (1.04–1.32)	1.49 (1.42–1.56)	1.04 (0.95–1.16)
Retail	1.21 (1.18–1.24)	1.18 (1.07–1.31)	1.28 (1.14–1.47)	1.38 (1.25–1.54)	1.10 (1.01–1.21)
Transportation or logistics	1.31 (1.26–1.36)	1.23 (1.10–1.41)	1.36 (1.27–1.45)	1.47 (1.35–1.60)	1.09 (1.04–1.15)
Not essential	1.12 (1.09–1.14)	1.14 (1.07–1.23)	1.26 (1.17–1.35)	1.28 (1.19–1.38)	0.98 (0.89–1.08)
Unemployed or missing	1.25 (1.22–1.28)	1.10 (1.03–1.18)	1.31 (1.24–1.40)	1.32 (1.23–1.43)	1.13 (1.06–1.21)

The relative excess is defined as the observed number of deaths divided by the expected number of deaths. The measure compares the pandemic to the counterfactual non-occurrence of the pandemic, as modeled using pre-pandemic data. The reference groups are the same groups of interest, under the counterfactual exposure.

**Table 3:** Per-capita excess mortality among California residents 18–65 years of age, by occupational sector and race/ethnicity, March through November 2020.

	All races	Asian	Black	Latino	White
All sectors	46 (43–49)	12 (4–21)	110 (93–126)	61 (51–70)	21 (9–33)
Facilities	83 (67–98)	39 (16–62)	252 (188–315)	83 (65–101)	70 (35–103)
Food or agriculture	75 (66–85)	35 (20–50)	128 (69–187)	97 (85–110)	36 (18–54)
Government or community	24 (20–29)	17 (5–29)	68 (34–100)	46 (39–53)	3 (-10–16)
Health or emergency	30 (27–34)	31 (28–35)	93 (60–125)	42 (29–54)	3 (-12–17)
Manufacturing	61 (57–64)	33 (17–49)	105 (30–179)	96 (86–105)	15 (-21–52)
Retail	38 (33–43)	25 (11–40)	76 (42–109)	41 (30–52)	30 (4–55)
Transportation or logistics	91 (81–102)	43 (20–65)	188 (153–222)	97 (80–114)	46 (22–70)
Not essential	17 (14–20)	11 (6–16)	81 (59–103)	30 (21–38)	-4 (-23–14)
Unemployed or missing	59 (52–65)	12 (4–21)	113 (90–136)	55 (42–67)	45 (22–68)

The per-capita excess mortality is defined as the observed number of deaths minus the expected number of deaths, divided by the population size, and multiplied by 100,000. The measure compares the pandemic to the counterfactual non-occurrence of the pandemic, as modeled using pre-pandemic data. The reference groups are the same groups of interest, under the counterfactual exposure.

Among occupations with 20 or more recorded COVID-19 deaths (Table 4), relative excess mortality was highest among sewing machine operators (59%), cooks (57%), miscellaneous agricultural workers (54%), butchers and other meat workers (52%), and couriers and messengers (52%).

**Table 4:** Excess mortality among Californians 18–65 years of age, by occupation: March through November 2020.

Description	Excess deaths	COVID-19 deaths	Per-capita excess	Relative excess
Sewing machine operators	70	73	200	1.59
Cooks	316	123	100	1.57
Miscellaneous agricultural workers	378	242	126	1.54
Butchers and other meat, poultry, and fish processing workers	40	20	164	1.52
Couriers and messengers	59	21	105	1.52
Production workers, all other	101	61	137	1.46
Metal workers and plastic workers, all other	35	34	546	1.43
Taxi drivers and chauffeurs	46	25	44	1.42
Bakers	34	23	89	1.40
Industrial truck and tractor operators	115	63	137	1.40
Packaging and filling machine operators and tenders	31	23	83	1.39
Construction laborers	756	269	227	1.38
Laborers and freight, stock, and material movers, hand	450	193	133	1.37
Miscellaneous assemblers and fabricators	82	40	76	1.37
Customer service representatives	160	47	46	1.36
Grounds maintenance workers	232	112	115	1.35
Stock clerks and order fillers	102	30	47	1.34
Security guards and gaming surveillance officers	204	86	120	1.34
First-line supervisors of housekeeping and janitorial workers	42	26	150	1.34
Maids and housekeeping cleaners	108	73	43	1.33
Nursing, psychiatric, and home health aides	121	54	67	1.32
Chefs and head cooks	143	58	168	1.32
Driver/sales workers and truck drivers	474	267	107	1.30
Social workers	54	20	49	1.29
Janitors and building cleaners	220	135	72	1.28

The table shows the 25 occupations with the most excess deaths, ranked by relative excess, and restricting to occcupations with 20 or more recorded COVID-19 deaths.

## Discussion

Our analysis of deaths among Californians between the ages of 18 and 65 shows that the pandemic’s effects on mortality have been greatest among essential workers, particularly those in the facilities, food/agriculture, manufacturing and transportation/logistics sectors. Excess mortality in high-risk occupational sectors was evident across all race and ethnic groups in stratified analyses, with notably high relative and per-capita excess in Latino and Black Californians.

Our findings are consistent with a small but growing body of literature demonstrating occupational risks for SARS-CoV-2 infection. For example, a study of the UK Biobank cohort found that essential workers, particularly healthcare workers, had high risks for COVID-19 [9]. Similarly, numerous studies have documented SARS-CoV-2 infection among healthcare workers [10]. Our study, however, is unique in examining excess mortality across multiple occupational sectors for all working-age decedents in California for the period of the pandemic, a comprehensive view that highlights the higher mortality risk across essential occupational sector. Though our work is in agreement with prior studies in finding pandemic-related risks among healthcare workers [10], it suggests that the risks are even higher in other sectors, such as food/agriculture and transportation/logistics.

This study is also among the first to examine deaths by both occupation and race/ethnicity. Occupational exposures have been postulated as an important contributor for disparities in excess mortality by race ethnicity, particularly because certain occupations require in-person work and because essential sectors also have a large fraction of low wage workers [4]. Both Black and Latino workers experienced substantial excess mortality during the pandemic. Latino workers experienced the greatest relative excess mortality while per-capita excess mortality was highest among Black workers. This pattern reflects the lower mortality among Latino workers compared to Black workers prior to the pandemic, so the larger per-capita excess among Black workers comprised a smaller percentage difference. Both per-capita and relative measures highlight mechanisms of disparities [11] and should be considered for targeted interventions to reduce COVID-19 related mortality. Disaggregating data by occupation and race/ethnicity also reveals important high risk occupational classifications among Asian and White working-age Californians who have lower overall excess mortality during the pandemic. Asian healthcare workers experienced very high relative excess, whereas in other race/ethnicity groups, healthcare workers experienced low relative excess. Such differences may reflect cross-sector differences in demographics; the large relative excess mortality among Asians in the health/emergency sector could be due to the relatively large number of Filipino Americans in nursing professions [12]. Variation by race/ethnicity may also reflect variability of risk within an occupation. For example, one job title may have higher risk within one sector than in another, or one manufacturing environment may be better ventilated or have better access to personal protective equipment than another. A recent study found, for example, that Black workers are more likely to be employed in occupations that frequently require close proximity to others [13]. Inequalities in risk may be exacerbated by underlying structural inequities, such as immigration status or poverty [14].

Our findings do not conclusively demonstrate that risks are entirely workplace related. Other factors may have led to excess mortality among certain occupational sectors, including crowded housing and access to healthcare. Disentanglement of such factors is outside the scope of the present study. However, we stress that whether transmission occurs at work is irrelevant to whether high-risk workers should be vaccinated: high-risk individuals will and should be protected by vaccination. Moreover, given the duration of time that individuals spend at work and documented weaknesses in workplace protection, workplace transmission seems likely. The public health response to the pandemic includes various policies and guidelines for minimizing transmission, including social-distance guidelines in public parks and financial support for infected individuals unable to safely quarantine; protections must absolutely exist at workplaces as well. In fact, non-workplace transmissions may in many cases be occupationally related; for example, the source for a household transmission may be a family member who became infected while on the job.

Though non-occupational risk factors may be relevant, it is clear that eliminating COVID-19 will require addressing occupational risks. In-person essential workers are unique in that they are not protected by shelter-in-place policies. Indeed, our study shows that excess mortality rose sharply in several essential sectors during the state’s first shelter-in-place period, from late March through May; these increases were not seen among those working in other sectors. Complementary policies are necessary to protect those who cannot work from home. These can and should include vaccination, personal protective equipment, clearly defined and strongly enforced safety protocols, easily accessible testing, generous sick leave policies, and appropriate responses to workplace safety violations. These protections have not been consistently applied. Prior to the pandemic, only about a third of workers in the lowest 10% of the wage distribution had access to paid sick leave [15]. And, the federal and state Occupational and Safety and Health Administration that sets and enforces these standards has been noted to be absent during the pandemic [16] (although some states, including California, have in the past few months adopted emergency temporary standards that specify workplace standards and allow for their enforcement).

We recognize limitations to our findings, including misclassification of occupation in death certificates due to coarse categories or inaccurate reports. The decedent’s primary occupation is typically reported by the next of kin who may not be able to precisely describe the work. The primary occupation, which is reported on the death certificate, may not match the most recent occupation, which is more likely to drive occupational risk. These limitations would in general attenuate apparent differences across occupational sectors but are unlikely to account for our primary results. As with other studies using similar methods nationally, the number of excess deaths in our study exceed the number of COVID-confirmed death. While other causes of death might also have risen during the pandemic, the temporal pattern of excess mortality among other factors suggest that undiagnosed COVID-19 may be a key contributor [17]. Regardless of the underlying cause, protecting working-age adults in occupations with higher deaths during the pandemic should be a focus of study and action.

Our study offers a powerful lens on the unjust impact of the COVID-19 pandemic on mortality of working-age adults in different occupations. Essential workers—especially those in the facilities, food/agriculture, manufacturing, and transportation/logistics sectors—face increased risks for pandemic-related mortality. Shutdown policies by definition do not protect essential workers and must be complemented with policies that will ensure safe workplaces, provide paid sick leave, and enable vaccination. If indeed these workers are essential, we must be swift and decisive in enacting measures that will treat their lives as such.

## References

[1] Weinberger DM, Chen J, Cohen T, et al. Estimation of excess deaths associated with the COVID-19 pandemic in the United States, March to May 2020. JAMA Intern Med. 2020;180(10):1336–1344. doi: 10.1001/jamainternmed.2020.3391 32609310 PMC7330834

[2] Woolf SH, Chapman DA, Sabo RT, Weinberger DM, Hill L, Taylor DDH. Excess deaths from COVID-19 and other causes, March-July 2020. JAMA. 2020;324(15):1562–1564. doi: 10.1001/jama.2020.19545 33044483 PMC7576405

[3] Rossen LM, Branum AM, Ahmad FB, Sutton P, Anderson RN. Excess deaths associated with COVID-19, by age and race and ethnicity—United States, January 26–October 3, 2020. MMWR Morb Mortal Wkly Rep. 2020;69:1522–1527. doi: 10.15585/mmwr.mm6942e2 33090978 PMC7583499

[4] Chen Y-H, Glymour MM, Catalano R, Fernandez A, Nguyen T, Kushel M, et al. Excess mortality in California during the COVID-19 pandemic, March-August, 2020. JAMA Intern Med. 2020;e207578. doi: 10.1001/jamainternmed.2020.7578 33346804 PMC7754079

[5] Bibbins-Domingo K. This time bust be different: disparities during the COVID-19 pandemic. Ann Intern Med. 2020;173(3):233–234. doi: 10.7326/M20-2247 32343767 PMC7192360

[6] Rodriguez-Diaz CE, Guilamo-Ramos V, Mena L, et al. Risk for COVID-19 infection and death among Latinos in the United States: examining heterogeneity in transmission dynamics. Ann Epidemiol. 2020;S1047-2797(20)30267-2. doi: 10.1016/j.annepidem.2020.07.007 32711053 PMC7375962

[7] State of California. Essential Workforce. https://covid19.ca.gov/essential-workforce/. Accessed October 1, 2020.

[8] Hyndman RJ, Athanasopoulos G. Forecasting: Principles and Practice. 2nd ed. Melbourne, Australia: OTexts; 2018.

[9] Mutambudzi M, Niedwiedz C, Macdonald EB, et al. Occupation and risk of severe COVID-19: prospective cohort study of 120 075 UK Biobank participants. Occup Environ Med. Published online December 9, 2020:oemed-2020-106731. doi: 10.1136/oemed-2020-106731 33298533 PMC7611715

[10] Gholami M, Fawad I, Shadan S, et al. COVID-19 and healthcare workers: a systematic review and metaanalysis. Int J Infect Dis. Published online January 2021. doi: 10.1016/j.ijid.2021.01.013 33444754 PMC7798435

[11] Harper S, Lynch J. Methods for Measuring Cancer Disparities: Using Data Relevant to Healthy People 2010 Cancer-Related Objectives. NCI Cancer Surveillance Monograph Series, Number 6. Bethesda, MD: National Cancer Institute, 2005. NIH Publication No. 05–5777.

[12] Wong T. Little noticed, Filipino Americans are dying of COVID-19 at an alarming rate. The Los Angeles Times. July 21, 2020.

[13] Hawkins D. Differential occupational risk for COVID-19 and other infection exposure according to race and ethnicity. Am J Ind Med. 2020;63(9):817–820. doi: 10.1002/ajim.23145 32539166 PMC7323065

[14] Riley AR, Chen Y-H, Matthay EC, et al. Excess deaths among Latino people in California during the COVID-19 pandemic. medRxiv. 2020;12.18.20248434. doi: 10.1101/2020.12.18.20248434 34307826 PMC8283318

[15] Michaels D, Wagner GR. Occupational safety and health administration (OSHA) and worker safety during the COVID-19 pandemic. JAMA. 2020;324(14):1389. doi: 10.1001/jama.2020.16343 32936212

[16] US Department of Labor. National Compensation Survey: Employee Benefits in the United States, March 2019. Washington, DC: US Department of Labor; 2019.

[17] Centers for Disease Control and Prevention. “Excess death” data point to pandemic’s true toll. Accessed April 16, 2021. https://www.cdc.gov/coronavirus/2019-ncov/cdcresponse/accomplishments/excess-death-data.html

